# Development and implementation of an aspiration pneumonia cause investigation algorithm

**DOI:** 10.1111/crj.13557

**Published:** 2022-11-14

**Authors:** Yuki Yoshimatsu, Kazunori Tobino, Omar Ortega, Hiroyuki Oda, Hiroaki Ota, Takafumi Kawabata, Yuri Hiramatsu, Yosuke Murakami, Pere Clavé

**Affiliations:** ^1^ Department of Respiratory Medicine Iizuka Hospital Fukuoka Japan; ^2^ Department of Physiology Hyogo College of Medicine Nishinomiya Japan; ^3^ Department of Respiratory Medicine Juntendo University Graduate School of Medicine Tokyo Japan; ^4^ Centro de Investigación Biomédica en Red de Enfermedades Hepáticas y Digestivas (Ciberehd) Instituto de Salud Carlos III Barcelona Spain; ^5^ Gastrointestinal Motility Laboratory, Department of Surgery, Hospital de Mataró, Consorci Sanitari del Maresme Universitat Autònoma de Barcelona Mataró Spain; ^6^ Department of General Medicine Iizuka Hospital Fukuoka Japan

**Keywords:** cause, deglutition, diagnosis, dysphagia, screening

## Abstract

The diagnostic criteria of aspiration pneumonia have not been established, and it remains an underdiagnosed entity. Diagnosis and cause investigation is essential in improving the management of aspiration pneumonia. The Japanese Respiratory Society Guidelines for the Management of Pneumonia in Adults (JRS Guidelines) show a list of risk factors for aspiration pneumonia. We developed an algorithm to aid physicians in evaluating these possible underlying factors and guide their management with a focus on aspiration pneumonia. The algorithm was developed based on the JRS Guidelines. The algorithm suggested dysphagia screening, pneumococcal and influenza vaccination, and other preventative measures for pneumonia. The algorithm was implemented in the acute setting of a general hospital among older patients admitted with pneumonia. Their outcomes were compared with a historical control group constituting similar patients from the previous year. Forty patients with pneumonia were assessed with the algorithm group, and 44 patients were included in the control group. In the algorithm group, significantly more cases (95.0% vs. 15.9%, *p* < 0.01) underwent early screening for a swallowing disorder. Two patients in the algorithm group were diagnosed with a new condition causing aspiration pneumonia, as opposed to none in the control group. Drugs with a potential risk for aspiration were identified and discontinued in 27.5% of the patients in the algorithm group and 4.5% in the control group. In conclusion, an aspiration pneumonia cause investigation algorithm translating the JRS guideline approach into practice enhanced the rate of swallow screening and preventative measures for aspiration.

## Graphical abstract

Aspiration pneumonia remains an underdiagnosed and unestablished diagnosis. We propose the use of an algorithm (developed based on the JRS guidelines) to enable adequate management of older adults with pneumonia. When implemented in an acute setting, the algorithm enhanced the diagnosis of causative conditions, swallow screening, and preventative measures.

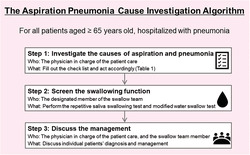

## 1 INTRODUCTION

Aspiration pneumonia has become an increasingly significant challenge, particularly since the demographic shift to a superaging society has increased the relative older population. It affects many patients and is a heavy burden on families and carers, in addition to healthcare professionals. Often diagnosed according to a characteristic clinical history (witnessed macroaspiration), predisposing risk factors, and chest radiography findings (such as consolidation in the lower lung fields),^1^ aspiration is said to account for as much as 80.1% of older adults (≥65 years) hospitalized with pneumonia.^2^ However, it is still a largely underdiagnosed entity in the everyday clinical setting. In fact, there have been cases that were originally thought to be mere lower respiratory tract infections, which were later found to be aspiration pneumonia due to a fatal underlying disease, such as brainstem hemorrhage,^3^ pharyngeal cancer,^4^ and diffuse idiopathic skeletal hyperostosis in the cervical spine.^5^ In addition, there is no established method in Japan for investigating the causative condition of aspiration. In our retrospective study, 30.7% of older adults admitted for aspiration pneumonia in which the cause was initially unknown were later diagnosed with a new causative condition of aspiration.^6^ It is suspected that more cases remain undiagnosed, as aspiration pneumonia is highly underdiagnosed, and its cause investigation is not performed uniformly. Appropriate treatment and preventative measures for possible aspiration pneumonia heavily depend on the correct diagnosis of the causative condition. The diagnosis of the causative condition enables treatment of the causative condition, prevention of aspiration, improvement of the quality of life, and informed decision making.^6^

The Japanese Respiratory Society Guidelines for the Management of Pneumonia in Adults (JRS Guidelines) show a list of risk factors that can put patients at risk of aspiration pneumonia.^7^ However, there is no guidance on how to screen for these factors. Other medical societies and guidelines have varied definitions and standpoints. Some hospitals have made original algorithms on how to screen for dysphagia or on how to level up dysphagia diets. However, the first challenge lies in how to evaluate the risk factors of aspiration and pneumonia, in order to differentiate between pneumonia and aspiration pneumonia. Furthermore, swallowing dysfunction is commonly seen in older adults and is a risk of pneumonia even when symptoms are subclinical.^8^ As the older population increases, more physicians are finding themselves in the role of managing pneumonia and its prevention, presenting an increasing need for an algorithm to effectively evaluate and eliminate the risks of aspiration in the elderly.

There are reports on how to suspect the risk of swallowing disorders in certain populations such as during surgery,^9^ postextubation,^10^ or associated with tube feeding.^11^, ^12^ Mylotte introduced tools that allow nursing home staff to suspect nursing‐home‐associated pneumonia and to differentiate between aspiration pneumonia and aspiration pneumonitis.^13^ This was focused more on the history and symptoms as it was aimed at nursing home staff to perform. A need for a clinical tool to help clinicians diagnose aspiration pneumonia has been stressed in many publications, including a recent literature review.^14^ Many professionals stress the importance of careful history taking,^6^, ^15^ whereas others suggest a more thorough process including screening and instrumental evaluation of swallowing.^16^ Prather et al. showed how to differentiate between multiple aspiration‐related lung diseases using imaging characteristics.^17^ However, no clear algorithm was found, which would guide nonspecialist clinicians in differentiating between pneumonia and aspiration pneumonia in older adults and investigating the cause of aspiration.

Therefore, we developed an algorithm based on the JRS guidelines to aid the steps into the diagnosis of aspiration pneumonia by investigating the possible underlying causes of aspiration and pneumonia. The main aim of this study was to implement this algorithm in the acute setting to investigate its feasibility and clinical effectiveness as a preliminary study. To our knowledge, this is the first study to develop and implement an aspiration pneumonia cause investigation algorithm.

## 2 METHODS

### 2.1 Development of the algorithm

**FIGURE 1:**
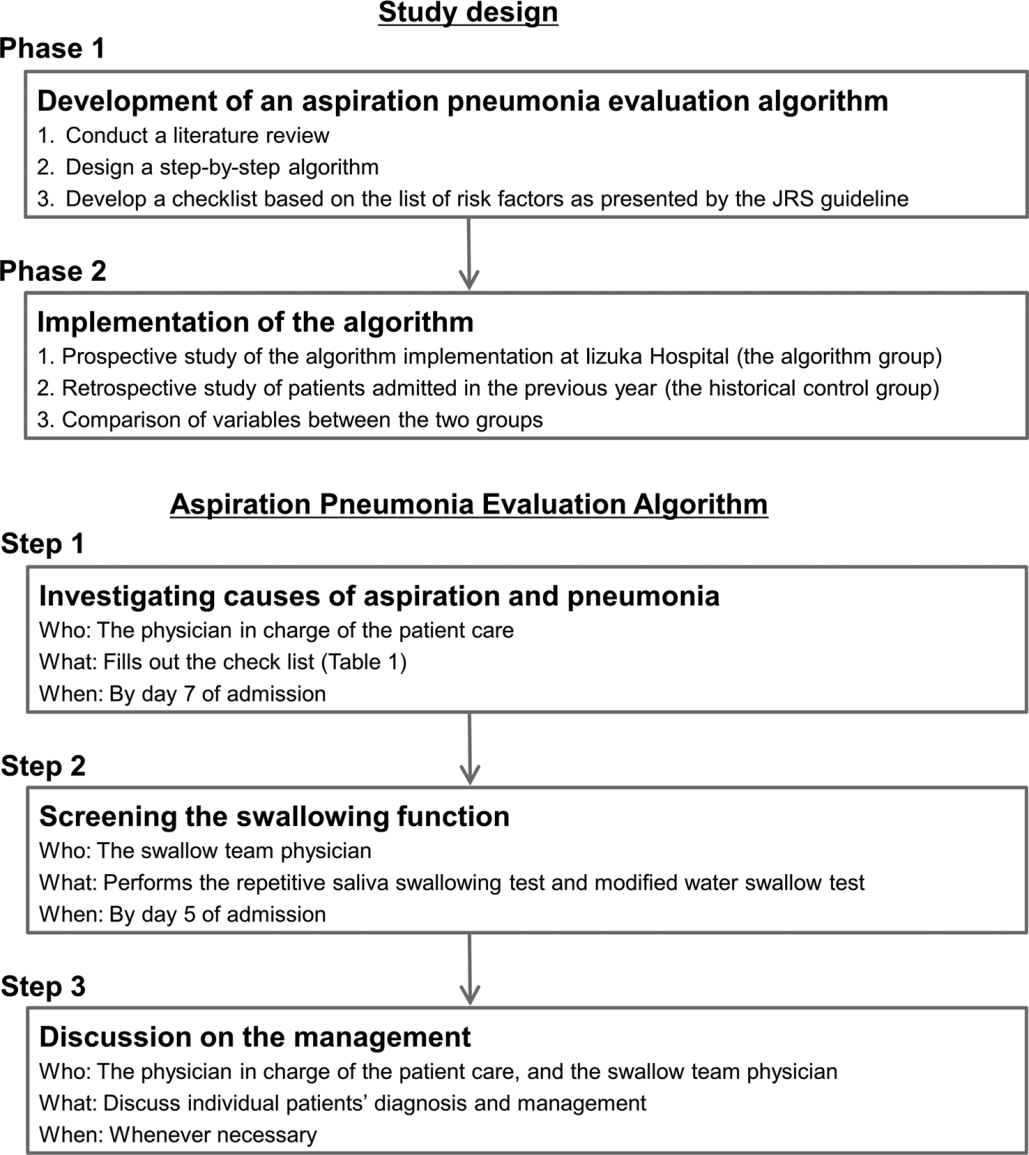
The aspiration pneumonia cause investigation algorithmThere were two steps to the algorithm. In the first step, the physician in charge of the patient care would investigate the cases of aspiration and pneumonia by filling out the checklist (Table 2). In step 2, the swallow team physician would screen the patient's swallowing function. When necessary, both physicians would discuss further investigation and management.

A step‐by‐step algorithm to evaluate the risks of aspiration and pneumonia was developed (Figure 1), based on the list of risk factors for aspiration and pneumonia, as presented by the JRS guidelines. The first step of the algorithm was to investigate the causes of aspiration and pneumonia using a checklist (Table 1). Literature was searched to identify a reliable and feasible way to screen for each entity in the JRS list. For example, the simplest criteria for stroke with the highest sensitivity is the Cincinnati Prehospital Stroke Scale, which consists of facial droop, arm drift, and speech problem.^18^, ^19^ Therefore, we listed these three signs on the algorithm and recommended to consider undergoing a head CT or MRI if any of these signs were newly found. Likewise, a simple four‐question cognitive test, the Mini‐Cog, is said to have similar sensitivity for detecting dementia as that of the MMSE, which is more time‐consuming.^20^, ^21^ Therefore, the Mini‐Cog was chosen as the criteria for detecting dementia. If positive, further intervention was recommended. In this manner, the algorithm was developed to show what conditions to find and how. As for iatrogenic causes (surgery, radiation therapy, and drugs), the details of which medical intervention could cause aspiration or pneumonia were listed, in addition to what steps could be taken to lower the risk of aspiration pneumonia. Finally, vaccination statuses for streptococcus pneumoniae and influenza virus were recommended to be checked and updated, as these vaccinations are reported to decrease the risk of pneumonia in older adults.^22^, ^23^

**TABLE 1:** Checklist for causes of aspiration and pneumonia

Risk factors for aspiration	Checklist and further management		
**Symptoms**			
Altered mental status	□ None	□ Known condition Details:______________	□ New or unconfirmed condition Investigate cause: hypoactive delirium, drug‐induced, epilepsy, etc.
Weakness and bedbound	□ None	□ Known condition Details:______________	□ New or unconfirmed condition Investigate cause: fracture, sarcopenia, neurological disorder, etc.
Oral and throat disorder	□ None	□ Known condition Details:______________	□ New or unconfirmed condition If there is hoarseness, wet voice, abnormal sensation in the throat, or tumor: consider further testing
Vomiting	□ None	□ Known condition Details:______________	□ New or unconfirmed condition Investigate cause (ultrasound, computed tomography, gastroscopy, etc.).
Nausea, heartburn, and feeling of chest congestion	□ None	□ Known condition Details:______________	□ New or unconfirmed condition Consider gastroesophageal reflux, hiatal hernia, tumor, candida, etc. Try a proton‐pump inhibitor or consider gastroscopy.
Temporomandibular joint dislocation	□ None	□ Yes: Consider dental consultation	
Oral dryness, hygiene issues, dental insufficiency, and unfitting denture	□ None	□ Yes: Consider dental consultation	
**Underlying conditions**			
Stroke and brain tumor	□ None	□ Known condition Details:______________	□ New or unconfirmed condition If there is facial droop, arm/leg numbness, or speech problem: head CT or MRI.
Parkinson's disease	□ None	□ Known condition	□ New or unconfirmed condition If there is resting tremor, slow movements, rigidity, or posture reflex disorder, consider further investigation.
Dementia	□ None	□ Known condition Details:______________	□ New or unconfirmed condition Perform Mini‐Cog. If positive, consider further intervention (including advance care planning).
Other neurological diseases	□ None	□ Known condition Details:______________	□ New or unconfirmed condition If there is unexplained muscle weakness etc., consider neurological consultation.
Chronic respiratory disease	□ None	□ Known condition Details:______________	□ New or unconfirmed condition Diagnose and consider treatment (chronic obstructive pulmonary disease, nontuberculous mycobacteria, diffuse panbronchiolitis, bronchiectasis, etc.).
Malnutrition	□ None	□ Known condition Details:______________	□ New or unconfirmed condition Consider investigating the cause and dietician consultation.
**Iatrogenic (non drug related)**			
**Tubes**			
Tracheostomy	□ None	□ Yes: Consider improving tube management.	
Nasogastric	□ None	□ Yes: Consider taking out, changing to a thinner tube, or stopping/changing nutrition.	
percutaneous endoscopic gastrostomy	□ None	□ Yes: Consider stopping/changing nutrition (amount, formula, speed).	
**Procedures**			
Surgery	□ None	□ Yes: oral, pharynx, larynx, stomach, esophagus.	
Radiation	□ None	□ Yes: oral, salivary glands, pharynx, larynx, esophagus.	
**Drug related**			
Dopamine antagonist	□ None	□ Yes: psychotic, antiemetic, or other (_________________)	
Muscle relaxant	□ None	□ Yes: sleep medication, anxiolytics, muscle relaxant, anti‐epileptic, or other (______________)	
Antitussive	□ None	□ Yes: opioid, or other (_________________)	
Oral dryness	□ None	□ Yes: antidepressant, diuretic, dysuria treatment, anti‐allergic, anti‐cholinergic, or other (_________________)	
Antacids	□ None	□ Yes: proton‐pump inhibitor, H2 blocker, or other (_________________)	
**Vaccination**			
Pneumococcal	□ None	□ Within 5 years	□ Never or more than 5 years ago: consider vaccination
Influenza	□ None	□ Current season	□ Not during this season: consider vaccination

As the second step of the algorithm, simple screening tests for dysphagia were to be performed within 5 days of admission. Two safe screening methods routinely used in Japan, namely, the repetitive saliva swallowing test (RSST)^24^, ^25^ and the modified Water Swallowing Test (mWST)^26^ were selected. Other tests were to be conducted when deemed necessary. It was recommended that the patient's attending physician and the swallow team physician discuss the patient's management whenever necessary. The algorithm was designed so that physicians could check for the risk factors of aspiration and pneumonia one by one in a unified manner, and in the event of a positive screening result, they were guided on the next steps to take (such as considering dental consultation or performing a gastroscopy). This would potentially lead to the prevention of future pneumonia. When a patient was found to have one or more risks of aspiration (Table 1) or if they screened positive on the dysphagia screening tests, it would suggest that this patient may have aspiration pneumonia. The algorithm was not intended to conclude whether the pneumonia was due to aspiration or not; rather, it was designed to guide physicians in the steps to take in the diagnostic process of pneumonia in older adults, with an aim to encourage consideration of aspiration.

### 2.2 Implementation of the algorithm

The algorithm was implemented in the acute setting as a prospective study to observe its feasibility and clinical effectiveness as a preliminary study. Ethical approval was provided by the Iizuka Hospital Ethics Committee as instituted by the Declaration of Helsinki (Number 20028). Patients aged 65 years and above who were admitted to Iizuka Hospital Department of Respiratory Medicine or General Medicine for the treatment of pneumonia from September 1 to November 30, 2020, were included. Patients were to be capable of giving informed consent or have an acceptable surrogate capable of giving consent on their behalf. Written consent was obtained from the patient or their surrogate. The exclusion criteria included the following groups who were prone to pneumonia from reasons other than aspiration: patients with an active malignancy, patients being treated with chemotherapy, systemic steroids, immunosuppressants, and patients admitted for the treatment of interstitial lung disease. Patients were recruited by the investigator and subinvestigators upon admission for pneumonia. Pneumonia was diagnosed according to the JRS Guidelines (which is defined by the presence of symptoms such as cough, sputum, fever, dyspnea and chest pain, and characteristic imaging abnormalities seen on chest X‐ray or CT). Patients were allowed to be withdrawn from the study for safety reasons, failure to adhere to protocol requirements, or upon their request.

The physician in charge of the inpatient care managed the patient with reference to the algorithm. The following variables were evaluated: patient background (age and sex), patient management (days of admission, swallow investigation, swallowing function, and the prevalence of a new cause of dysphagia being identified), and prevention of future aspiration (discontinuance of risky medications, dentist and dental hygienist involvement, pneumococcal vaccination, and influenza vaccination).

These variables were compared with the historical control group: patients admitted in the previous year (September 1 to November 30, 2019). The control group was retrospectively selected to be similar to that of the algorithm group, in order to increase overall validity. The criteria were as follows: all patients aged 65 years and above, admitted for the treatment of pneumonia (not interstitial lung disease), and without any disease or treatment predisposing them to infection (i.e., chemotherapy, immunosuppressants, and systemic steroids). Informed consent for patients in the control group was waived by the Ethics Committee of Iizuka Hospital due to its retrospective nature.

### 2.3 Statistical analyses

Descriptive statistics for baseline data were presented as the percentage, mean, and standard deviation. Differences between the two groups were examined using Mann–Whitney's U test, the chi‐square test, or Fischer's exact test. A *p*‐value of <0.05 was considered to indicate a statistically significant difference. All data analyses were carried out using the JMP Pro software program (ver. 15; SAS Institute, Cary, NC, USA).

## 3 RESULTS

A total of 40 patients were prospectively included in the algorithm group. The control group consisted of 44 patients. Patient demographic data are shown in Table 2. There was no significant difference between the two groups regarding patient background. There were no adverse events related to the algorithm. There were no withdrawals.

**TABLE 2:** Patient's characteristics

		Algorithm group *N* = 40	Control group *N* = 44	*p* value
Background	Age: median (range)	80 (65–97)	82 (65–92)	0.24
	Male/Female	26/14	27/17	0.73
Management	Hospitalized days: median (range)	14 (6–47)	21.5 (2–63)	0.15
	Swallow screening performed by day 5	38	7	<0.01^a^
	Abnormal results in swallow screening	22	7	<0.01^a^
	Diagnosis of a new cause of dysphagia	2	0	0.22
Prevention	Discontinuance of risky drugs	11	2	<0.01^a^
	Dentist or oral hygienist intervention	5	4	0.73

^a^ Fischer's exact test.

Regarding the management of the patient, early screening for swallowing disorder was performed statistically significantly more often in the algorithm group than in the control group (38 [95.0%] vs. 7 [15.9%], *p* < 0.01, Fischer's exact test). The reason two patients did not undergo swallow screening within 5 days of admission was that they were still intubated at that time. Subsequently, significantly more patients were found to have swallowing disorders (22 [55.0%] in the algorithm group vs. 7 [15.9%] in the control group, *p* < 0.01, Fischer's exact test). The algorithm group showed a tendency to have shorter hospital stays compared to the control group (16 days vs. 21.5 days, *p* = 0.15, Mann–Whitney's U test). In the algorithm group, two patients were diagnosed with a new condition causing aspiration pneumonia: esophageal hiatal hernia in one case and severe Alzheimer's disease in the other. There was no such case in the control group.

With regard to preventative measures for aspiration, drugs with a potential risk for aspiration were discontinued in 11 cases (27.5%) in the algorithm group, as opposed to two cases (4.5%) in the control group (*p* < 0.01, Fischer's exact test). These included benzodiazepines, sedatives, antipsychotics, cough suppressants, proton‐pump inhibitors, anticholinergics, and opioids. There was no adverse effect due to the discontinuance of these agents. There was no significant difference in the rate of consultation with the dentist and dental hygienist (five in the algorithm group vs. four in the control group, *p* = 0.73, Fischer's exact test).

The final diagnosis on the discharge summary was aspiration pneumonia in 17 cases (42.5%) in the algorithm group and 11 cases (25%) in the control group. In the algorithm, group there were eight cases, which were originally thought to be nonaspiration pneumonia on admission and were diagnosed as aspiration pneumonia after implementing the algorithm. In the control group, there were two cases, which were originally thought to be nonaspiration pneumonia on admission and were subsequently decided otherwise.

## 4 DISCUSSIONS

Despite the desperate need for guidance in the initial evaluation of possible aspiration pneumonia, no established criteria could be found in the literature. Therefore, an algorithm was developed based on the list of risk factors for aspiration and pneumonia presented in the JRS Guidelines. To the best of our knowledge, this was the first algorithm developed to guide physicians in putting the JRS Guidelines into practice and to improve the cause investigation of pneumonia in older adults.

The 3‐month implementation study revealed many positive effects of the algorithm. First, significantly more cases underwent early swallowing screening (38/40 patients underwent RSST and mWST by day 5), and significantly more cases were found to have abnormal screening results. This is mainly thought to be due to good compliance with the algorithm. In the current guidelines, it is not considered a routine procedure to screen for swallowing disorders in older adults. Therefore, whether screening is performed or not depends on the physician's awareness of swallowing disorders. Typically, in an average acute setting, swallowing is screened by the speech therapist if deemed necessary by the physician. Oftentimes, choking on water or during meals is what triggers physicians and nurses to consult a speech therapist. This may explain the high prevalence of abnormal screening results in those who were screened in the control group (100%) while also implying the presence of undiagnosed dysphagic patients in the unscreened population. As most pneumonia in older dults is caused by silent aspiration,^2^ swallowing screening is recommended even when choking is not witnessed. The algorithm enabled a swallow screening to take place within the first 5 days of hospitalization, making physicians aware of possible risks of aspiration that may have been unnoticed if the algorithm were not implemented. Swallow assessment with a videofluoroscopy or endoscopy is not a routine exam in the current Japanese practice (especially in the acute setting); therefore, this was not considered a necessity in the algorithm.

Two cases in the algorithm group were diagnosed with a new condition that was unknown on admission. The diagnosis of the causative condition of aspiration leads to the treatment of the cause, prevention of pneumonia, and better management of the patient.^6^ Step 1 of the algorithm, which was a checklist of the patient's condition, aided the screening process of the risk factors presented in the JRS Guidelines.

Regarding preventative measures for future pneumonia, drugs that may put patients at risk of aspiration were discontinued in 27.5% of cases in the algorithm group, significantly more than the control group (4.5%). Therefore, using a checklist may improve the physician's adherence to these recommendations, and hence lead to better patient outcomes.

The diagnosis of aspiration pneumonia is not established well. Many patients are diagnosed as having pneumonia, and not aspiration pneumonia, as it is difficult to distinguish between the two, especially during the primary assessment. When the possibility of aspiration is left unnoticed, necessary preventative measures cannot be taken, putting patients at risk of recurrent pneumonia, choking, malnutrition, and weakening. As we suggested in our previous study, it is necessary to shift our concentration from diagnosing whether a patient has aspiration pneumonia or not, to a more realistic approach with an emphasis on taking the necessary history and physical examination, and screening swallowing function in risky populations. This approach is also in line with suggestions in current guidelines. Our algorithm, designed to collect information on the possibility of a swallowing disorder and any predisposing conditions, embodies this approach.

The implementation of the algorithm caused no adverse events, and it was feasible within the daily clinical practice of an acute setting, in this preliminary study. Physicians ranging from their first year to those far more experienced all adhered to the protocol without difficulty.

There are several limitations associated with this study. First, items on the checklist were strictly limited to those presented in the list on the JRS guidelines; therefore, not all conditions that could cause aspiration pneumonia were listed. This was intended to maintain the validity of the development of the list. Second, this was a short‐term trial at a single tertiary care hospital and the subjects were a heterogenic population. The situation may be different in other circumstances. Third, not all suggestions by the algorithm were followed; for example, dental consultation or further examination were not performed in some cases even when indicated by the algorithm. However, as the algorithm was intended to be a simple guidance and not an enforcement, it is natural that not all cases follow it meticulously. Nevertheless, further consideration on how to effectively communicate the intention of the algorithm is awaited. In addition, the control group includes patients from 1 year prior to the algorithm group. This was to ensure that the control group had no influence from the algorithm. Although the guidelines and the majority of the physicians had not changed and the inclusion and exclusion criteria were also kept similar for both groups, there may have been changes in the management of pneumonia in relation to the recent COVID‐19 pandemic. However, in the rural area that Iizuka Hospital covers, during the time of the study, the pandemic's effect on daily clinical practice was minimal. We did not define aspiration pneumonia or aim to differentiate clearly between aspiration and nonaspiration pneumonia. Rather, this study was intended to aid the physician in detecting risk factors of aspiration and managing the patient well. We believe this is of importance in the current clinical setting in which the clear diagnostic criteria of aspiration pneumonia remain uncertain. Future studies on the definitive diagnosis of aspiration pneumonia are awaited.

We believe that these data are meaningful as an initial exploratory study. Future studies to assess the effect of the algorithm on clinical outcomes are likewise awaited. To improve the algorithm, an implementation study involving a larger population of patients and other healthcare professionals, and a proper reliability study may be of value.

An algorithm to search for aspiration risk in elderly patients with pneumonia was developed to put the JRS guideline approach into practice. Its implementation in the acute setting enhanced the rate of swallow screening, cause investigation, and preventative measures.

## CONFLICTS OF INTEREST

All authors have no conflicts of interest to disclose.

## ETHICAL STATEMENT

Ethical approval was provided by the Iizuka Hospital Ethics Committee as instituted by the Declaration of Helsinki (Number 20028). Patients were to be capable of giving informed consent or have an acceptable surrogate capable of giving consent on their behalf. For the implementation group, written consent was obtained from the patient or their surrogate. For the historical control group, informed consent was waived by the Ethics Committee of Iizuka Hospital due to its retrospective nature.

## AUTHOR CONTRIBUTIONS

Yuki Yoshimatsu designed and performed the study, collected data, and wrote the paper. Kazunori Tobino supervised the study design, performed the study, analyzed the data, and produced the figures. Omar Ortega and Pere Clavé supervised the algorithm development, edited the paper, and were in charge of overall supervision. Hiroyuki Oda, Hiroaki Ota, Takafumi Kawabata, Yuri Hiramatsu, and Yosuke Murakami collected the data and edited the manuscript.

## Data Availability

The data that support the findings of this study are available from the corresponding author, Yuki Yoshimatsu, upon reasonable request.
